# Gut Microbiota-Derived Components and Metabolites in the Progression of Non-Alcoholic Fatty Liver Disease (NAFLD)

**DOI:** 10.3390/nu11081712

**Published:** 2019-07-25

**Authors:** Yun Ji, Yue Yin, Ziru Li, Weizhen Zhang

**Affiliations:** 1 Department of Physiology and Pathophysiology, Peking University Health Science Center, Beijing 100191, China; 2 Department of Surgery, University of Michigan Medical Center, Ann Arbor, MI 48109-0346, USA

**Keywords:** gut microbiota, metabolite, liver disease, metabolism

## Abstract

Human gut microbiota has been increasingly recognized as a pivotal determinant of non-alcoholic fatty liver disease (NAFLD). Apart from the changes in the composition of gut microbiota, the components and metabolites derived from intestinal microbiota have emerged as key factors in modulating the pathological process of NAFLD. Compelling evidences have revealed that gut microbiota generates a variety of bioactive substances that interact with the host liver cells through the portal vein. These substances include the components derived from bacteria such as lipopolysaccharides, peptidoglycan, DNA, and extracellular vesicles, as well as the metabolites ranging from short-chain fatty acids, indole and its derivatives, trimethylamine, secondary bile acids, to carotenoids and phenolic compounds. The mechanisms underlying the hepatic responses to the bioactive substances from gut bacteria have been associated with the regulation of glycolipid metabolism, immune signaling response, and redox homeostasis. Illuminating the interplay between the unique factors produced from gut microbiome and the liver will provide a novel therapeutical target for NAFLD. The current review highlights the recent advances on the mechanisms by which the key ingredients and metabolites from gut microbiota modulate the development and progression of NAFLD.

## 1. Introduction

Non-alcoholic fatty liver disease (NAFLD), a spectrum of liver disease, includes simple steatosis (NAFL), non-alcoholic steatohepatitis (NASH), fibrosis and cirrhosis. NAFLD has become the leading liver disease, with a prevalence of 22–29% in adults worldwide [1]. NAFLD is associated with metabolic syndrome which is characterized by centripetal obesity, insulin resistance, hypertension, hyperlipidemia, and dyslipidemia [2]. As of today, the intricate pathogenesis of NAFLD remains largely unknown. The “multiple hit” hypothesis has been proposed to supersede the outdated “two-hit” hypothesis as the major cause of the initiation and progression of NAFLD [3]. The “multiple hit” hypothesis highlights the importance of gut microbiome, insulin resistance, and adipokines secreted from the adipose tissues, which consequently leads to lipotoxicity, oxidative stress, mitochondrial dysfunction, and inflammation in hepatic tissue [3].

It has been estimated that 10 times more microorganisms (above 10^14^) are present inside the gut than the number of human cells [4]. These microorganisms are associated with the regulation of host metabolism, immunity, and diseases. Gut microbiome has drawn extensive attention as it plays a critical role in the development and progression of NAFLD via the gut-liver axis [5]. Long term intake of unhealthy diet (e.g., rich in saturated fat or fructose) initiates dysbiosis of gut microbiota which in turn leads to disruption in barrier function and immune homeostasis. Gut microbiota or the components and metabolites derived from bacteria are carried to the liver through the portal vein [6]. A variety of immune cells (e.g., Kupffer cells and hepatic stellate cells) resided in the liver copes with the gut-derived pro-inflammatory factors such as lipopolysaccharide, peptidoglycan, and lipoteichoic acid (LTA). The over-activated immune cells induced by the products generated from bacteria may result in more severe liver damage, inflammation, and fibrosis, thus accelerating the development of NAFLD. On the other hand, metabolites such as short-chain fatty acids (SCFAs), bile acids, tryptophan metabolites, carotenoids, and phenolic compounds from gut bacteria may ameliorate the inflammatory responses, oxidative damage, and lipogenesis in the liver tissue. Therefore, intestinal microbes are considered as the key element regulating the pathological process of NAFLD. Exploring the signaling pathways of the gut bacteria-derived factors on the liver tissue will provide novel therapeutic targets and strategy for NAFLD. The current article will summarize the functional mechanisms by which the gut microbial components and metabolites alter the progression of NAFLD.

## 2. Lipopolysaccharides

Lipopolysaccharides (LPS, also known as endotoxins), the major outer membrane component of gram-negative bacteria, has been well known to be implicated in the activation of the host innate immune system. The chronic low grade inflammation triggered by LPS is known as a pivotal factor for the progression of NAFLD. Previous study has demonstrated that treatment with antibiotics (e.g., polymyxin B) targeting gram-negative bacteria efficiently reduced tumor necrosis factor (TNF) production and plasma LPS levels, leading to the reversal of hepatic steatosis [7]. Elevation of LPS levels in serum has been reported in both NAFLD patients and animals [8,9]. Toll-like receptor 4 (TLR4), a pattern recognition receptor for LPS and multiple free fatty acids, is widely expressed in liver cell types including hepatocytes, Kupffer cells, and stellate cells [10]. Activation of TLR4 induced by LPS results in the secretion of inflammatory cytokines (e.g., IL-6, IL-1β, and TNF-α) and chemokines from Kupffer cells, leading to hepatic damage and NASH [11,12]. By contrast, mice carrying a TLR4 mutation exhibit resistance to LPS and high-fat diet-induced liver steatosis [13]. In addition to TLR4, LPS binding protein (LBP) and cluster of differentiation 14 (CD14) are also involved in the recognition of LPS. LBP is a polypeptide of 50 kDa primarily synthesized by liver and exists in blood as a form of glycosylated protein [14]. A clinical study has found an increase of LBP, a carrier of LPS, among patients with NAFL or NASH [15]. Besides, LBP is correlated with insulin resistance and dyslipidaemia [16]. Since free fatty acids are coupled with chronic low grade inflammation and steatohepatitis, loss of LBP may attenuate the progression of NAFLD. Indeed, it has been observed in HFD-induced NAFLD animal models that LBP knockout mice display improved lipid metabolism and alleviation of multiple pathologic features of NAFLD [17]. This observation suggests that LBP is an indispensable factor for the development of NAFLD. In contrast, it has been noted that a high level of LBP appears to limit the inflammatory responses triggered by LPS, likely through delivering LPS to lipoproteins or promoting silent uptake of LPS [18,19,20]. CD14 is a myeloid membrane glycoprotein that functions as a pattern recognition receptor for the complexes of LPS and LBP. CD14 exists in two forms: membrane CD14 (mCD14) and soluble CD14 (sCD14, also known as presepsin). LPS induces a cleavage of mCD14, leading to the subsequent release of presepsin into the circulation [21]. Depletion of CD14 reduces lipid and macrophage content in liver tissues and attenuates liver steatosis in diet-induced obese mice [22]. The clinical trial indicates that serum presepsin levels can be regarded as a biomarker for predicting the severity of NASH [23]. The mechanism by which LPS produced by gut microbiota contributes to the occurrence and development of NAFLD involves the intestinal barrier dysfunction. Several lines of evidence have shown that increments of circulating LPS impairs intestinal barrier function and causes subsequent increases in intestinal permeability. This occurs through the TLR-4-dependent up-regulation of CD14 and MLCK (Myosin light chain kinase), and activation of IRAK-4 (IL-1R-associated kinase 4) [24,25,26]. More importantly, LPS that enters into the liver through the portal vein blood activates Kupffer cells and stellate cells via TLR4, which promote hepatic inflammation and fibrosis respectively. In addition, activation of NLRP3 inflammasome induced by LPS has recently been shown to be linked with the progression of NASH. The detail review can be found in the article by Wan et al. [27].

## 3. Peptidoglycan

Peptidoglycan (PGN) is an inflexible envelope encompassing the cytoplasmic layer of most bacterial species [28]. It is a unique and necessary bacterial component that provides the bacterial cell wall with stiffness and structure. Compared to the single thin layer surrounded by an outer membrane in Gram-negative bacteria, PGN is presented as a thick exposed layer in Gram-positive bacteria, in combination with lipoteichoic acid (LTA) [29]. It has long been known that PGN from bacteria promotes an inflammatory response. In mice with a high fat diet, the abundance of Firmicutes (the major gram-positive bacteria) increases. This alteration is associated with the increment of Toll-like receptor 2 (TLR2) ligands including PGN, lipoprotein, and LTA [30,31]. The role of TLR2 in the pathophysiology of NASH has been controversial depending on the model of NASH. In high fat diet-induced obese mice, TLR2 deficiency has been shown to be resistant to insulin resistance, hepatic steatosis, and tissue inflammation [32,33]. Conversely, in mice fed with a diet deficient in methionine and choline (MCDD), TLR2 knockout exacerbates MCDD-induced nonalcoholic steatohepatitis. In a human consuming a high fat diet, impaired TLR2 response observed is partly responsible for the increased risk of obesity [34]. The sub-structures of PGN, such as meso-diaminopimelic acid PGN (meso-DAP PGN) and muramyl dipeptide PGN (MDP PGN), can mediate the generation of pro-inflammatory cytokines through nuclear factor-κB (NF-κB)/mitogen-activated protein kinase (MAPK) dependent activation of NOD1 (Nucleotide Binding Oligomerization Domain Containing 1) and NOD2 (Nucleotide Binding Oligomerization Domain Containing 2) [35,36,37]. It has been proposed that NOD1 senses excess nutrients by altering the intestinal microbiota and enhancing peptidoglycan translocation, since NOD1 can identify peptidoglycan from the intestinal microbiota [38]. Supporting the function of the NOD1/2 signaling in the development of NAFLD, mice with a dual knockout of NOD1 and NOD2 are resistant to lipid accumulation and inflammation in the liver caused by a high fat diet [39]. In short, PGN-mediated inflammatory lesions in the hepatic tissue are primarily associated with the signaling pathways of TLR2, NOD1, and NOD2. It should be noted that TLR2 is also known as a key ligand for other bacterial components such as LTA and lipoproteins. In rodents, gram-positive bacteria component LTA actuates hyperlipidemia which occurs as a consequence of increased lipolysis of excessive hepatic triglyceride [40].

## 4. Bacterial DNA

Bacterial DNAs play a vital role in the progression of NASH by the direct activation of immune cells including macrophages, NK cells, B cells, and dendritic cells. Bacterial DNA is trafficked into endolysosomal endosomes through endocytosis, in which it activates Toll-like receptor 9 (TLR9) to elicit inflammatory signaling in immune cells [41]. The sensing of bacterial DNA by TLR9 in immune cells initiates the activation of NF-κB / MAPK, followed by the secretion of IL-12 and TNF-α [42]. Alternatively, TLR9 results in the activation of IκB kinase α (IKKα) which interacts with microtubule-associated protein 1A/1B-light chain 3 (LC3) and the phosphorylation of IFN regulatory factor 7 (IRF7), leading to the release of type I interferon (IFN) [43,44]. TLR9 responses through species-specific CpG motif recognition to bacterial DNA [45]. Recent evidence has shown that TLR9 is augmented in human and mouse NASH. This receptor is essential for the chemotaxis of M1-macrophages and neutrophils [46]. Removal of TLR9 mitigates liver inflammation in experimental NASH mice [46]. Consistently, a TLR9 antagonist treatment protects mice fed a high fat diet from NASH [47]. Furthermore, it has been observed in choline-deficient amino acid-defined (CDAA) diet-triggered NASH model that the production of IL-1β in Kupffer cells induced by TLR9-MyD88 cascades accelerates the progress of NASH [48].

## 5. Extracellular Vesicles

A large variety of bacterial molecules, including nucleic acids, proteins, phospholipids, glycolipids, and polysaccharide, are present in microbiota-derived extracellular vesicles (EVs) [49]. EVs play important biological roles not only in the survival of bacteria through transferring virulence factors and the despoilment of nutrients, but also in the interaction between the host and the bacterial components or metabolites that regulate numerous signaling pathways in the host cells [50]. Bacterial EVs that enter systemic circulation, as nanosize membrane particles, can trigger multiple metabolic cascades and immunological responses in various organs. For example, recent progress reported by Tulkens et al. [51] has shown that patients with intestinal barrier dysfunction demonstrate increased systemic levels of LPS-positive bacterial extracellular vesicles. This observation suggests that the endotoxin is able to alter the biological function of the host via its presence on EVs. Emerging evidences have shown that host cells carry LPS from EVs into the cytosol by the signaling of TLR4-TRIF (TIR domain-containing adaptor-inducing interferon-β)-GBPs (guanylate-binding proteins) [52]. Other receptors mediating EVs-induced immune responses in host cells include TLR2, NOD1, and NOD2 [53,54]. As innate immune is a key mechanism mediating the inflammation and other pathological progress in NAFLD, EVs may regulate NAFLD through transferring the contents into Kupffer cells, stellate cells, and hepatocytes. Although the precise protein inside the EVs are largely unknown, the proteomics approach has revealed novel interaction pathways between bacterial effector protein and hepatic cells underlying the physiological and pathological functions of EVs [55,56]. Short RNA (sRNA) in EVs can be released to alter biological functions of the host [57]. Hence, EVs may have the potential to regulate NAFLD through sRNA-mediated epigenetic mechanism.

## 6. Short-Chain Fatty Acids

Short-chain fatty acids (SCFAs) are volatile fatty acids (acetate, propionate, and butyrate) mainly generated through the fermentation of soluble dietary fibers and nondigestible carbohydrates by gut microbes in the large bowel [58]. The producers of acetic acid and propionate for the most part are the Bacteroidetes, whereas butyrate is principally delivered by the Firmicutes [59]. Gut microbiota has been observed to be engaged with the modulation of NAFLD by means of their metabolites SCFAs. SCFAs regulate the metabolism and immune functions of liver tissue mainly through the inhibition of histone deacetylases or the activation of G-protein coupling receptors including GPR41, GPR43, GPR109a, and OLFR78. GPR41 is known to be expressed in adipose, intestinal, spleen, pancreas, peripheral blood, and nervous tissues, while GPR43 is present in the immune, liver, intestinal, and adipose tissues [60,61,62]. All three SCFAs, especially acetate, can be recognized by GPR43. Mice exhibit increased GPR43 mRNA abundance in liver specific manner in response to a high fat diet feeding [63], suggesting a unique role of GPR43 in obesity-related liver diseases. Besides, propionate is a selective agonist for GPR41, while butyrate acts as an activator for GPR41 and GPR109a. Particularly, acetate and propionate serve as ligands for OLFR78 which is abundantly expressed in the colon and kidney [64,65]. Depending on SCFA, both GPR41 and GPR43 participate in the regulation of energy homeostasis. A lack of GPR41 increases the body fat mass of mice, suggesting that SCFAs promote energy expenditure and prevent obesity via activation of GPR41 [66]. Deficiency of GPR43 renders mice resistant to the increase in liver weight and triglycerides content induced by HFD to at least in part through an increase in energy consumption [67]. Inconsistently, results from Kimura et al. [68] have found that mice lacking the GPR43 gene develop obesity even on a regular diet. The activation of GPR43 results in the suppression of insulin signaling and thus prevents the uptake and utilization of energy. A recent study by Rau et al. [69] has found a higher level of SCFAs produced from gut bacteria in NAFLD patients. The increase of acetate and propionate maintains low degree inflammation through their impact on the circulating immune cellular system. However, the anti-inflammatory properties and the suppression role in hepatic lipogenesis and lipid accumulation of acetate and propionate have been observed in numerous previous studies [70,71,72]. Importantly, compelling data from animal models has shown that butyrate attenuates steatohepatitis through the modulation of gut microbiota, intestinal barrier function, the up-regulation of glucagon-like peptide-1 receptor (GLP-1R) expression and down-regulation of inflammatory signaling as well as oxidative damage in the liver [73,74,75,76]. It should be noted that histone acetylation regulates gene expression through the alteration in the structure of the nucleosomes or furnishing protein binding signaling [77]. The addition of acetylate groups is catalyzed by histone acetyltransferases (HATs) while the elimination of acetylate groups is by histone deacetylases (HDACs). SCFAs from gut microbiota has been widely known as HDAC inhibitors to regulate immune homeostasis and hepatic lipid metabolism, suggesting an epigenetic mechanism by which SCFAs regulate host metabolism.

## 7. Indole and Its Derivatives

Tryptophan can be converted into several molecules, including indole and its derivatives, by both intestinal gram-positive and gram-negative bacteria present in the gut. Indole, which is produced through the catalysis of tryptophan by bacterial tryptophanase, has attracted much attention because of its beneficial effects on intestinal function of the host. Numerous bacterial species including the genera of Prevotella, Bacteroides, Fusobacterium, and Escherichia possess the capacity to degrade tryptophan into indole by tryptophanase [78,79,80,81]. Indole functions to enhance tight junction of epithelial cells and mitigates inflammatory responses and injury in the gut [82,83]. Additionally, indole modulates glucagon-like peptide-1 (GLP-1) secretion in colonic L cells via increasing calcium influx and reducing the decomposition rate of GLP-1 [84]. Recently, Beaumont et al. [85] have demonstrated that mice receiving indole display resistance to liver inflammation and metabolic alternations of cholesterol induced by LPS. This observation suggests that indole may improve inflammatory disorder in the liver. Other indole derivatives that have received widespread attentions include indole-3-aldehyde (IAld), indole-3-acetic acid (IAA), and indole-3-propionic acid (IPA). These molecules exert vital biological functions through the action on the nuclear receptors in host cells. IAld is mainly synthesized from indole pyruvate under the catalysis of aromatic amino acid aminotransferase (ArAT). As a ligand of aryl hydrocarbon receptor (AhR), IAld induces the production of IL-22, thereby providing a protection against candidiasis and mucosal damage [86]. Moreover, recent evidence from the human colon cell line has demonstrated that IAld induces the expression of IL-10R1, indicating an IL-10 signaling-dependent anti-inflammatory mechanism of IAld [87]. Emerging researches have revealed a reduction in the level of IAA in individuals with metabolic syndrome. For instance, results from Natividad et al. [88] have found that fecal samples of obese people demonstrate lower levels of IAA than those of non-obese people. Also, a reduction in the concentration of IAA in the colon content has been observed in mice fed with a high fat diet, which exhibits glucose intolerance and hepatic steatosis. Another study has reported a substantial deprivation of IAA in the liver and cecum (3 and 10-fold, respectively) of mice exposed to a high fat diet compared with those fed a normal diet [89]. This study also demonstrates that IAA dose dependently reduces the induction of pro-inflammatory cytokines including TNF-α, MCP-1, and IL-1β by LPS, leading to a reduction in the synthesis of FFAs and palmitate in macrophage cell line. Besides, IAA alleviates the lipogenesis mediated by cytokine and free fatty acids via its direct action on hepatocytes in an AhR-dependent manner. The evidences above suggest a protective role of IAA against NAFLD through acting on both macrophages and hepatocytes. IPA, which serves as a ligand for pregnane X receptor (PXR), has also been shown to suppress intestinal pro-inflammatory cytokines and strengthen the barrier function [90]. As a free radical scavenger, IPA is known to protect against oxidative stress-induced damage in brain, neuron, and hepatic microsomal membranes [91,92,93]. Intriguingly, recent study uncovers that IPA improves glucose metabolism by reducing the levels of blood glucose and plasma insulin [94]. Therefore, IPA may possess the therapeutic potential for metabolize dysfunction related to insulin resistance.

## 8. Bile Acids

Bile acids, which are synthesized from cholesterol in the liver and stored in the bile bladder, regulate multiple physiological and pathological processes. Bile acids not only facilitate the absorption of lipids in the intestine, but also function to modulate glucose and lipid metabolism [95]. The therapeutic value of bile acids has been known in various diseases, including neurological disorders, fatty liver, liver inflammation, gallstones, primary biliary cirrhosis, pancreatitis, and inflammatory bowel disease [96,97,98,99,100,101]. Gut microbiota converts the primary bile acids including cholic acid (CA) and chenodeoxycholic acid (CDCA) in the distal small intestine and colon of human beings into secondary bile acids such as deoxycholic acid (DCA), lithocholic acid (LCA), and ursodeoxycholic acid (UDCA) [102]. Compared with primary bile acids, secondary bile acids are more conducive to the selection of intestinal bacteria. In addition to their direct antimicrobial properties, bile acids indirectly involve in farnesoid X receptor (FXR)-mediated antimicrobial defense [103,104]. FXR is a member of the nuclear hormonal receptor family that widely exists in ileum and the liver, and regulates gene expression in diverse metabolic pathways. Modulation of FXR signaling has emerged as a potential strategy for prevention and treatment of the fatty liver and relevant disturbance of metabolism [105]. FXR has been reported to reduce fatty acid and triglyceride synthesis in the liver through down-regulating the expression of LXR and SREBP-1C [106]. FXR-deficient mice exhibit a reduction in the tolerance for glucose and decrease in the sensitivity to insulin [107]. In contrast, FXR activation by cholic acid (CA) reduces glucose levels by inhibiting expression of multiple genes related to gluconeogenesis in the liver [107]. Reduction of phosphoenolpyruvate carboxykinase (PEPCK) expression induced by small heterodimer partner (SHP) further suggests the crucial role of FXR in the regulation of glucose metabolism [108]. Aside from FXR, Takeda-G-protein-receptor-5 (TGR5) is another classic receptor for bile acids. In hepatic tissue, TGR5 is expressed in Kupffer and endothelial cells and functions to modulate liver inflammation and glucose metabolism, and to improve insulin sensitivity. TGR5 mitigates inflammatory response through the inhibition of NF-κB signaling and cytokines generation in macrophages [109]. In addition, TGR5 possesses a pivotal role in controlling the intestinal GLP-1 release from L cells and thus in maintaining the homeostasis of glucose [110]. Interestingly, supplementation of bile acids to mice facilitates energy expenditure in brown adipose tissue in TGR5-dependent manner [111]. These evidences suggest that TGR5 is functionally active in the regulation of inflammation, glucose metabolism and energy balance.

## 9. Trimethylamine

Trimethylamine (TMA) produced from the intestinal microbiota through the catabolism of dietary choline, phosphatidylcholine, betaine, and carnitine, enters into the liver via the portal vein [112]. Trimethylamine-N-oxide (TMAO), an oxidative product of TMA catalyzed by flavin-containing monooxygenases (FMO) in the liver, has been considered as a novel biomarker for early metabolic syndrome [113]. TMAO affects the development of NAFLD through multiple pathways. Firstly, the elevation of blood TMAO predicts an increase in TMA production, indirectly reflecting the changes in the metabolism of choline and phosphatidylcholine. In particular, deficiency of choline may hinder the synthesis and secretion of very low-density lipoprotein, thus leading to hepatic accumulation of triglycerides and fatty degeneration [114]. Secondly, the expression of hepatic cytochrome P450 family 7 subfamily A member 1 (CYP7A1) gene, which encodes an endoplasmic reticulum membrane protein that catalyzes the conversion of cholesterol to bile acids, is positively associated with the serum level of TMAO in NAFLD patients [115]. However, mice supplemented with TMAO in normal physiological conditions show a reduction in bile acid. This is attributed to the decrease in the expression of bile acid synthetic enzymes and transporters in the liver [116]. Thus, TMAO may modulate NAFLD via the regulation of bile acid metabolism and transport. Finally, gut microbiota-mediated TMA/FMO3/TMAO pathway modulates insulin resistance, glycolipid metabolism, cholesterol homeostasis, and hepatic inflammation [117,118,119], thereby affecting hepatic triglyceride accumulation and liver steatosis.

## 10. Carotenoids and Phenolic Compounds

The importance of dietary phytonutrients including carotenoids and polyphenols in amelioration of NAFLD has been highlighted by numerous human and animal studies [120,121,122,123,124,125]. Both carotenoids and polyphenols are discovered as secondary plant metabolites that possess potential anti-oxidant and anti-inflammatory properties. Carotenoids are a family of fat-soluble tetraterpenoids compounds that present as pigments in vegetables and fruits [126], while polyphenols are known as molecules that contain more than one hydroxyl group linked to benzene rings [127]. Although the sources of carotenoids and polyphenols are mostly from plants, gut microbiota-derived contributions are of great concern. The bioaccessibility and bioavailability of dietary carotenoids and polyphenols, which have known to be improved by gut microbiota, may determine the extent of their benefits on human health [128,129]. A growing appreciation has emerged for the role of gut microflora in facilitating the generation of carotenoids and phenols, through the degradation of intestinal content into simple molecules by microbial enzymes. Interestingly, comparative genomics have revealed four major phylogenetic lineages of microbiota with the capacity of carotenoid biosynthesis [130]. In addition, an in vitro gut fermentation experiment uncovered the formation of phenolic metabolites of microbe in response to water-extractable dietary fiber [131]. Hence, the microbiota present in the human gut brings about an increase in the concentrations of carotenoids and phenolic compounds in the circulatory system and thus the target tissues. The precise mechanisms of the protective roles of carotenoids and phenolic compounds in NAFLD are not fully elucidated, however, apart from the direct roles as free radical scavengers and anti-inflammatory molecules, carotenoids and phenolic compounds have been shown to function as agonists for AHR and PXR, thereby maintaining gut homeostasis, normal glucose metabolism, and resistance to inflammatory disorders [132,133,134,135,136]. Further research is required to provide the mechanism underlying the defensive effects of carotenoids and phenolic compounds on NAFLD in specific receptor-dependent manner.

## 11. Conclusions

**Figure 1:**
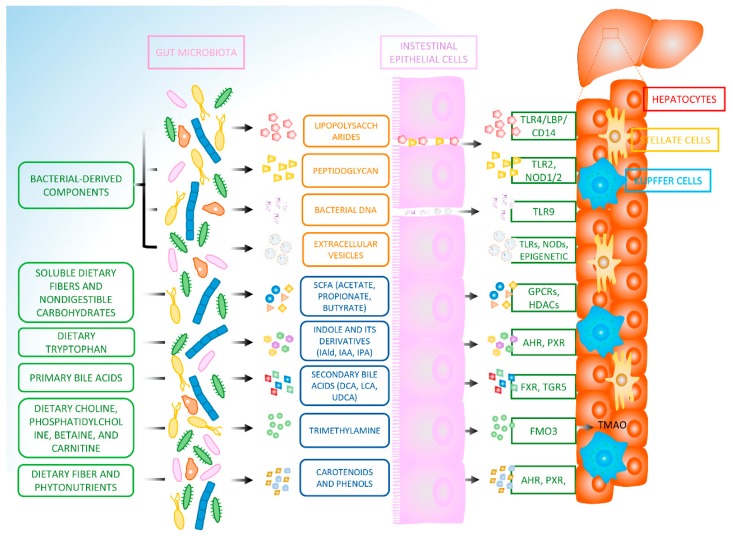
The mechanisms that connect gut microbiome-derived ingredients and metabolites with non-alcoholic fatty liver disease (NAFLD). Impaired intestinal mucosal barrier function leads to leakage of bacterial components (lipopolysaccharides, peptidoglycan, DNA, and extracellular vesicles) from the gut into the portal vein that provides blood to different liver cell types (Kupffer cells, hepatic stellate cells, and hepatocytes). These bacterial components result in activation of the corresponding toll-like receptors (TLR2, TLR4, TLR9) or NOD-like receptors (NOD1, NOD2) and the subsequent signaling pathway. The short RNA (sRNA) inside the extracellular vesicles serve as epigenetic regulators in gene expressions. The intestinal microbiome can be subjected to soluble dietary fibers and nondigestible carbohydrates fermentation which produce SCFAs (acetate, propionate, and butyrate). Short-chain fatty acids work through binding to their receptors (GPCRs) or by inhibiting the activity of histone deacetylases (HDACs). Dietary tryptophan is metabolized to indole and its derivatives (indole-3-aldehyde, indole-3-acetic acid, and indole-3-pyruvic acid) by the tryptophanase generated from specific species of gut microbiome, followed with the activation of AHR and PXR signaling in the liver tissue via the portal vein. Primary bile acids (cholic acid (CA) and chenodeoxycholic acid (CDCA)) can be metabolized to secondary bile acids (deoxycholic acid (DCA), lithocholic acid (LCA), and ursodeoxycholic acid (UDCA)) which regulates FXR and TGR5 signaling in the liver. The bacteria that resides in the gut also metabolizes choline into trimethylamine which is oxidized to trimethylamine-N-oxide (TMAO) by hepatic flavin-containing monooxygenases 3 (FMO3). Dietary components (e.g., fiber and phytonutrients) provide substrates for microbiota-derived carotenoids and phenols which act on hepatic tissue directly or via AHR and PXR activation.

The gut microbiota is closely related to NAFLD and functions through delivering their own ingredients or metabolites. Bacterial endotoxin, peptidoglycan, DNA, and extracellular vesicles-induced inflammation may accelerate the development of NAFLD and the onset of NASH. Patients suffering from NAFLD appear intestinal dysbiosis and impaired intestinal barrier function, which in turn aggravates the uptake of bacterial component into the hepatic tissue and thus the immune responses. The key metabolites generated from gut microbiota, including short-chain fatty acids, secondary bile acids, indole and its derivatives, trimethylamine, carotenoids, and phenolic compounds, serve as regulators in host metabolism, immune cell systems, and redox homeostasis, thereby fundamentally altering the progression of NAFLD. Despite considerable progress being reported on the interaction between gut microbes and the liver diseases, the underlying mechanisms have not yet been fully elucidated. The mechanisms that clarify the association of the ingredients and metabolites derived from gut microbiome with NAFLD has been shown in Figure 1, according to the existing reports. The targeted application of microbial-derived factors requires a comprehensive description of their cellular receptors and potential signaling pathways on the host, which needs more extensive clinical and experimental research. Exploring the mechanisms whereby the components and metabolites generated from gut microbiota regulate host cells is in favor of the manipulation of susceptibility to liver diseases. Targeting gut microbiota-derived factors and the relevant host cell signaling will provide novel strategies for the intervention of NAFLD.

## Abbreviations

AhR	Aryl hydrocarbon receptor
EV	Extracellular vesicle
FXR	Farnesoid X receptor
GPCR	G-protein coupling receptor
HDAC	Histone deacetylase
HFD	high fat diet
IAA	Indole-3-acetic acid
IAld	Indole-3-aldehyde
IPA	Indole-3-propionic acid
LBP	LPS binding protein
LPS	Lipopolysaccharide
NAFLD	Non-alcoholic fatty liver disease
NASH	Non-alcoholic steatohepatitis
NOD1	Nucleotide Binding Oligomerization Domain Containing 1
PGN	Peptidoglycan
PXR	Pregnane X receptor
SCFA	Short-chain fatty acid
TGR5	Takeda-G-protein-receptor-5
TLR	Toll-like receptor
TMA	Trimethylamine
TMAO	Trimethylamine-N-oxide
